# Knockdown of GINS2 inhibits proliferation and promotes apoptosis through the p53/GADD45A pathway in non-small-cell lung cancer

**DOI:** 10.1042/BSR20193949

**Published:** 2020-04-03

**Authors:** Feng Chi, Zhou Wang, Yuzhu Li, Ning Chang

**Affiliations:** 1 Department of Thoracic Surgery, Shandong Provincial Hospital Affiliated to Shandong University, Ji’nan 250021, Shandong, China; 2 Intensive Care Unit, Yantai Affiliated Hospital of Binzhou Medical University, Yantai 264003, Shandong, China; 3 Pharmacy Intravenous Admixture Services, Yantai Affiliated Hospital of Binzhou Medical University, Yantai 264003, Shandong, China; 4 Department of Critical Medicine, Yantai Affiliated Hospital of Binzhou Medical University, Yantai 264003, Shandong, China

**Keywords:** Apoptosis, GADD45A, GINS2, NSCLC, p53, Proliferation

## Abstract

Lung cancer is a malignant tumour type with the highest morbidity and mortality, and non-small-cell lung cancer (NSCLC) is the most common pathological type. GINS complex subunit 2 (GINS2) is a member of the GINS family and is closely related to DNA replication and damage, participates in cell cycle regulation and plays a key role in cell proliferation and apoptosis. In the present study, we aimed to explore the role and underlying molecular mechanism of GINS2 in the development of NSCLC*.* The results showed that GINS2 is significantly increased in NSCLC tissues and cell lines. Knockdown of GINS2 significantly decreases cell proliferation, causing G_2_/M phase cell cycle arrest. Knockdown of GINS2 reverses the effect of nocodazole on the levels of cyclin-dependent kinase 1 (CDK1) and cyclin-B1. Meanwhile, knockdown of GINS2 significantly elevates the apoptosis rate and apoptosis-related protein Bax and decreases Bcl-2. In addition, GINS2 knockdown induces an increase in the levels of p53 and growth arrest and DNA damage 45A (GADD45A). Co-transfection with GINS2-siRNA and siRNA against p53 (p53-siRNA) or co-transfection with GINS2-siRNA and siRNA against GADD45A (GADD45A-siRNA) partially reverses the effects of GINS2 knockdown on cell proliferation and apoptosis. Taken together, these results indicate that GINS2 knockdown down-regulates cell proliferation, induces G2/M phase cell cycle arrest and increases apoptosis, possibly through the p53/GADD45A pathway.

## Background

Lung cancer is one of the malignant tumours that seriously endangers human health and has the highest morbidity and mortality in the world. Non-small-cell lung cancer (NSCLC) accounts for almost 80% of lung cancer deaths [1–3]. Most NSCLCs are diagnosed at an advanced stage, and thus the window of opportunity for surgical treatment is missed [4,5]. Chemotherapy plays an important role in the treatment of advanced lung cancer at present, but the effect of chemotherapy is still not ideal. Moreover, the quality of life of patients is seriously affected by the serious and inevitable side effects of chemotherapy [6,7]. In recent years, molecular targeted therapy for NSCLC has become a hot topic. Several new drugs specific to these key targets have achieved considerable clinical efficacy [8]. Due to its selection of suitable genetic mutations in patients and drug resistance in patients, it has become the bottleneck of its wide application [9]. Thus, the search for new targeted drugs that mediate the initiation and progression of NSCLC and can be used for molecular targeted therapies is urgent and of great interest.

GINS complex subunit 2 (GINS2), located in region 2 and zone 4 of the long arm of chromosome 16, with a full length of 1196 bp, is also known as Psf2, which forms the GINS complex with GINS4, GINS1 and GINS3 [10]. In eukaryotic cells, the Go-Ichi-Ni-San (GINS) family is closely associated with the occurrence and initiation of cell DNA damage and replication, participates in cell cycle regulation and has an important effect on cell proliferation and apoptosis [11,12]. Several studies have shown that GINS2 is abnormally expressed in many tumours, such as breast cancer [13], leukaemia [14], lung adenocarcinoma [15], and so on. Rantala et al. indicated that silencing the *GINS2* gene can inhibit cell growth and activity in human breast cancer cell lines [16]. In addition, Gao et al. found that knockdown of GINS2 could inhibit cell growth and promote apoptosis in the leukaemia K562 NB4 cell line [17]. These findings all suggest that GINS2 plays an important role in cancer progression. However, the significance of GINS2 in lung cancer has not been investigated.

In the present study, we evaluated the expression and function of GINS2 in NSCLC. Our results indicated that the expression of GINS2 was significantly down-regulated in tumour tissues, and the NSCLC cell lines A549 and H460. Loss-of-function experiments revealed that GINS2 was closely related to cell proliferation and apoptosis via the p53/growth arrest and DNA damage 45A (GADD45A) signalling pathway.

## Materials and methods

### Tissue collection

Lung adenocarcinoma tissues and adjacent normal tissues (distance from tumour margin ≥5 cm) were obtained with informed consent from 37 consecutive patients undergoing NSCLC resection surgery between July 2009 and March 2010 at Shandong Provincial Hospital affiliated to Shandong University. The specimens were immediately placed in liquid nitrogen and stored in a −80°C freezer. Lung adenocarcinoma tissues and adjacent normal tissues were confirmed by pathology. All patients signed an informed consent before surgery, and the present study was approved by the ethics committee of Shandong Provincial Hospital affiliated to Shandong University.

### Cell culture

The human lung adenocarcinoma cell line A549 was preserved by our laboratory. The non-tumorigenic lung cell line Gekko Lung-1 and the lung adenocarcinoma cell line H460 were purchased from the cell bank of the Shanghai Institute of Life Sciences, Chinese Academy of Sciences. Cells were cultured in DMEM (Sigma–Aldrich, St. Louis, MO, U.S.A.) supplemented with 10% foetal bovine serum, penicillin at a concentration of 1 × 10^6^ U/l and streptomycin at a concentration of 0.1 g/l. Aseptic culture was conducted in an incubator with a CO_2_ concentration of 5% and a temperature of 37°C, and the solution was substituted at intervals of 2 days. Cells in the logarithmic growth phase were taken for experiments.

### Transfection

SiRNA against GINS2 (GINS2-siRNA), siRNA against p53 (p53-siRNA), siRNA against GADD45A (GADD45A-siRNA) and siRNA negative control (NG) were purchased from Shanghai Gene Chem (Shanghai, China). On the day of transfection, A549 and H460 cells in the logarithmic growth stage were uniformly inoculated into six-well plates, with the number of cells being 2 × 10^5^ in each well. Plasmids were transfected into cells using Lipofectamine 2000 transfection reagent (Thermo Fisher Scientific, Wilmington, DE), according to the manufacturer’s instructions.

### RNA extraction and reverse transcription-quantitative PCR (RT-qPCR)

Total RNA was extracted by using the TRIzol method, and then the RNA was reverse-transcribed into cDNA. The expression of GINS2 was quantified by using the PrimeScript® RT reagent Kit (TaKaRa, Dalian, China), and GAPDH was used as the internal control. The reaction system for qPCR was a total of 50 μl containing 2 μl cDNA, 1 μl reverse primer, 1 μl forward primer, 25 μl premix Taq and 21 μl sterile water. The reaction conditions were as follows: 95°C pre-denaturation for 30 s, then 40 cycles of 95°C for 5 s and 55°C for 30 s. The relative gene expression was calculated by using the 2^−ΔΔ*C*_T_^ method. The primers for GINS2 and GAPDH were as follows: GINS2 F: 5′-GCTGGCGATTAACCTGAAAC-3′, GINS2 R: 5′-TTCCTTTC

GTTCATGATCCC-3′, GAPDH F: 5′-TGACTTCAACAGCGACACCCA-3′, GAPDH

R: 5′-CACCCTGTTGCTGTAGCCAAA-3′.

### Cell proliferation

Cells from the interference group, NG group and untreated (UN) group were inoculated uniformly into 96-well plates with 5 × 10^3^ cells in each well. The proliferation of A549 and H460 was assessed with the Cell Counting Kit-8 (CCK-8) assay kit (Dojindo Molecular Technologies, Shanghai, China). After 4 days of continuous measurement, a cell growth curve was constructed, and the test results were analysed with SPSS software. The experiment was performed in triplicate.

### Cell cycle distribution

Cells from the interference group, NG group and UN group were collected and washed three times with precooled PBS and then treated with 70% ethanol for 6 h. The annexin V/propidine iodide (PI) Apoptosis Detection kit was used to assess the cell cycle according to the manufacturer’s instructions (Beckman Coulter, Brea, CA).

### Annexin V-FITC/PI dual staining

The stably screened cells after transfection and the cells of each control group were centrifuged at 1000×***g*** for 5 min, and then the supernatant was discarded, and the cells were collected. The cells (5.0 × 10^4^) were stained with 100 µl binding buffer containing 5 µl Annexin V-APC fully mixed re-suspension. The reaction was carried out at room temperature for 10 min and centrifuged at 2000×***g*** for 5 min. The supernatant was further discarded, and the cells were resuspended by adding 190 µl of conjugated solution. Finally, 10 µl PI dye was added, mixed well at a low temperature and shielded from light for inspection. The apoptosis rate was measured by flow cytometry (FCM) .

### Western blot assay

Whole cell lysates were extracted from the interference, NG and UN groups. The protein content of each cell extract was determined by the BCA method (Pierce; Thermo Fisher Scientific, Inc.), and proteins were separated on 12% SDS/PAGE gels, then transferred to polyvinylidene fluoride (PVDF) membranes. Membranes were blocked at room temperature for 3 h and then incubated overnight at 4°C with primary antibody against GINS2, cyclin-B1, cyclin-dependent kinase 1 (CDK1), p-p53, histone, GADD45A, Bax, Bcl-2 and GAPDH (Cell Signaling Technology, Inc.), followed by horseradish peroxidase–conjugated secondary antibody (Pierce, Rockford, IL) for 1 h at room temperature. Histone was used as an internal reference of p53, and GAPDH was used as an internal reference of GINS2, cyclin-B1, CDK1, GADD45A, Bax and Bcl-2. The experiment was performed in triplicate.

### Statistical analysis

SPSS 22.0 software was applied to analyze the data. The experiment was repeated more than three times. The data are represented as the mean ± SD. One-way analysis of variance (one-way ANOVA) was used to compare the groups and the Student’s *t* test was used for pairwise comparison between groups. *P*<0.05 was considered to indicate statistical significance.

## Results

### GINS2 reveals higher expression in NSCLC tissues and cell lines

**Figure 1:**
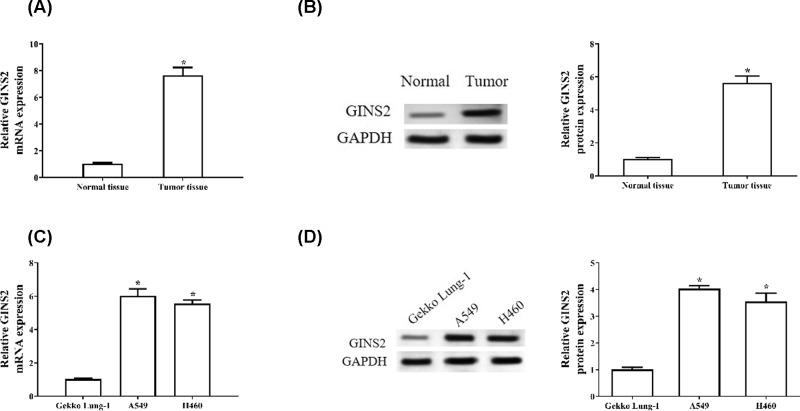
The relative expression level of GINS2 in NSCLC tissue and cell lines (**A**) The mRNA and (**B**) protein levels of GINS2 in NSCLC tissue and normal tissue were measured through RT-qPCR and Western blotting. (**C**) The levels of GINS2 mRNA and (**D**) protein in the normal lung cell line Gekko Lung-1, and the NSCLC cell lines A549 and H460 were checked by RT-qPCR and Western blot. *P<0.05 vs control group.

*GINS2* gene expression was evaluated using RT-qPCR and Western blot assays. The results showed that the expression levels of GINS2 mRNA and protein are increased significantly in NSCLC tissue compared with normal tissue (*P*<0.05, Figure 1A,B). We also measured the expression of GINS2 in a non-tumorigenic lung cell line (Gekko Lung-1) and two human NSCLC cell lines (A549 and H460). As shown in Figure 1C,D, GINS2 expression levels are significantly up-regulated in A549 and H460 cell lines compared with Gekko Lung-1 cell lines.

### Knockdown of GINS2 inhibits cell proliferation in A549 and H460 cells

**Figure 2:**
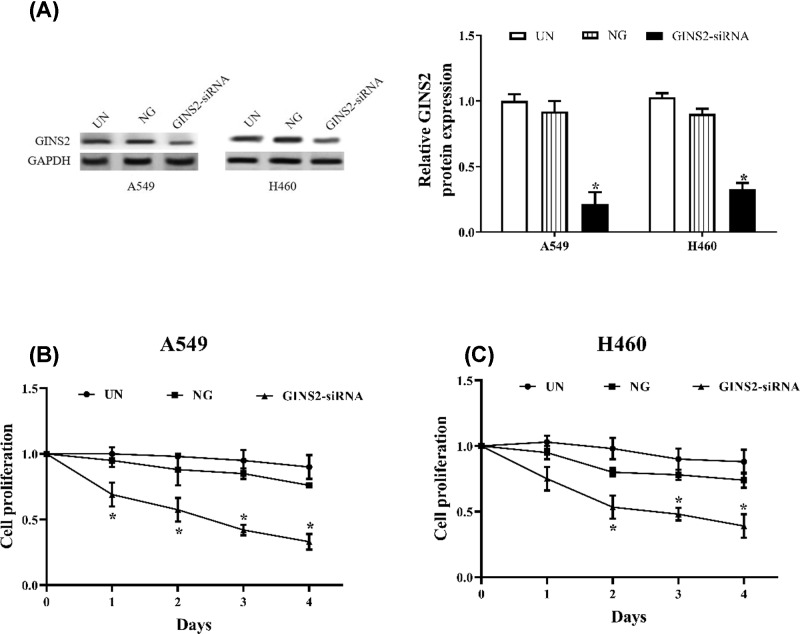
GINS2 regulates cell proliferation in A549 and H460 cells (**A**) The transfection efficiency of GINS2-siRNA was checked by Western blotting. (**B,C**) Cell proliferation was examined by CKK-8 in A549 and H460 cells. *P<0.05, compared with the NG group.

To determine the functional significance of GINS2 in NSCLC, a loss-of-function approach was employed in cultured A549 and H460 cells by transfection of interference plasmid. Cell proliferation was detected by CCK-8 assay after GINS2-siRNA transfection for 0, 1, 2, 3 and 4 days. With the down-regulation of GINS2 by transfection of GINS2-siRNA in A549 and H460 cells (Figure 2A), cell proliferation was decreased significantly compared with the NC group (*P*<0.05), except after transfection of GINS2-siRNA for 1 day in H460 cells, and the significance gradually increased with time. No significant difference was found between the NG group and the UN group (Figure 2B,C).

### Knockdown of GINS2 causes G_2_/M phase cell cycle arrest in A549 and H460 cells

**Figure 3:**
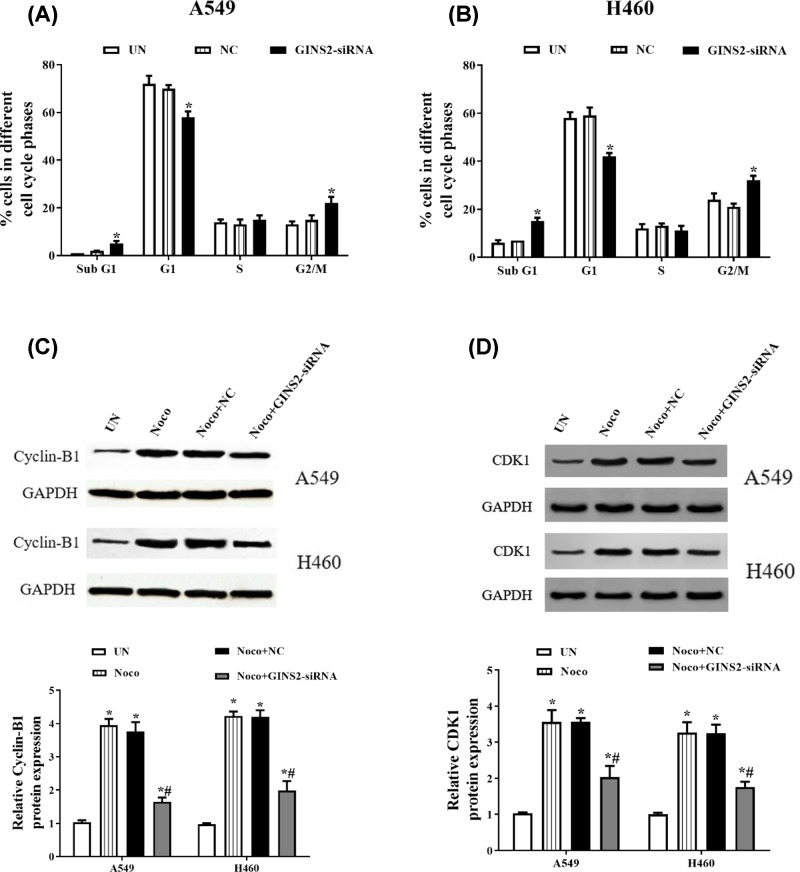
The effect of GINS2-siRNA on cell cycle arrest in A549 and H460 cells Detection of cell cycles after stable transfection for 48 h. (**A,B**) Flow cytometry detects the cell cycle in A549 and H460 cells. *P<0.05, compared with the NG group (**C**) Cell-cycle-related protein cyclin-B1 and (**D**) the level of CDK1 were measured by Western blotting. *P<0.05, compared with the Noco group. Abbreviation: Noco, nocodazole-treated group.

In order to further study the role of GINS2 silencing in the cell proliferation of NSCLC, flow cytometry was used to detect the cycle changes in A549 and H460 cells. The results indicated that the percent of G_2_/M is significantly increased, and sub-G_1_ and G_1_ phases are down-regulated in the GINS2-siRNA group compared with the NC group (*P*<0.05, Figure 3A,B). These results showed that the cell cycle is blocked in the G_2_/M phase. In addition, treatment with nocodazole significantly increases the expression levels of CDK1 and cyclinB1 protein, yet GINS2 knockdown obviously reverses the effect of nocodazole on the expression levels of CDK1 and cyclinB1 protein (*P*<0.05, Figure 3C,D).

### GINS2 knockdown induces apoptosis in A549 and H460 cells

**Figure 4:**
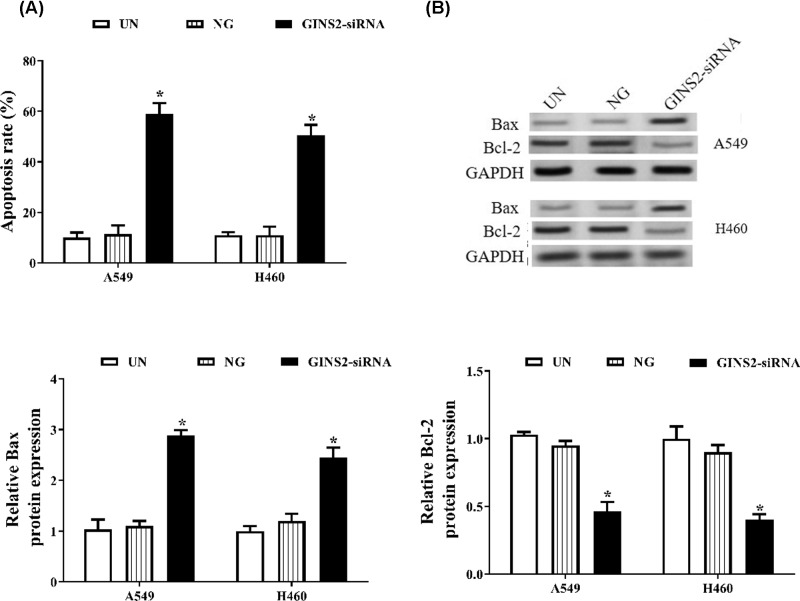
GINS2-siRNA promotes apoptosis in A549 and H460 cells (**A**) Detection of apoptosis rate after stable transfection for 48 h by flow cytometry. (**B**) Apoptosis proteins were measured by Western blotting. *P<0.05, compared with the NG group.

In the present study, we further explored the role of GINS2 silencing in the apoptosis of lung cancer cells. The results indicated that the apoptosis rate in A549 and H460 cells is increased remarkably after knockdown of GINS2 expression (*P*<0.05, Figure 4A). The pro-apoptosis-related protein Bax is significantly up-regulated, and the anti-apoptosis protein Bcl-2 is significantly reduced after knockdown of GINS2 expression in A549 and H460 cells (*P*<0.05, Figure 4B).

### Knockdown of GINS2 expression inhibits proliferation and promotes apoptosis via promotion of p53/GADD45A signalling

**Figure 5:**
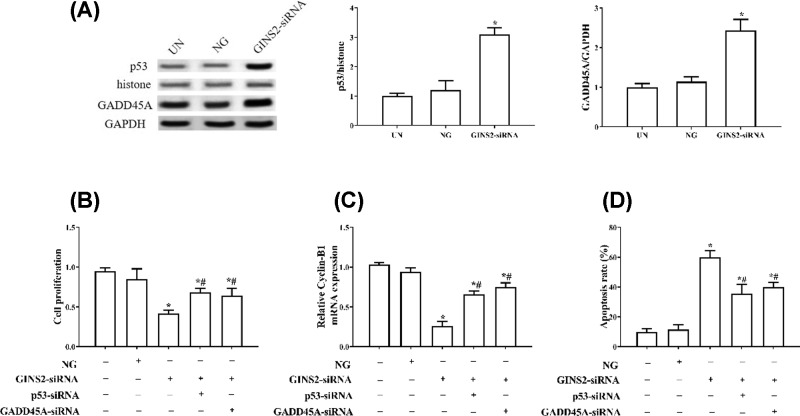
The effect of GINS2 knockdown on proliferation and apoptosis was attenuated by repressing p53 or GADD45A A549 cells transfected with GINS2-siRNA, NC, GINS2-siRNA + p53-siRNA, or GINS2-siRNA + GADD45A-siRNA were challenged for 48 h. (**A**) Western blotting measures the levels of p53 and GADD45A. (**B**) Cell proliferation was detected by CKK-8 assay. (**C**) Cell cycle-related protein cyclin-B1 was measured by Western blotting. (**D**) The cell apoptosis was determined by flow cytometry. Apoptosis-related proteins were checked by Western blot. *P<0.05, compared with the NG group; ^#^*P*<0.05, compared with the GINS2-siRNA group.

To explore the mechanism by which GINS2 affects cell proliferation and apoptosis in lung cancer cells, we further examined the p53/GADD45A axis. After the expression of GINS2 is inhibited, the levels of p53 and GADD45A protein are significantly up-regulated in A549 cells (*P*<0.05, Figure 5A). As shown in Figure 5B,C, co-transfection of GINS2-siRNA and p53-siRNA or co-transfection of GINS2-siRNA and GADD45A-siRNA increases cell proliferation and the level of cyclinB1 compared with the GINS2-siRNA group (*P*<0.05). In addition, co-transfection of GINS2-siRNA and p53-siRNA or co-transfection of GINS2-siRNA and GADD45A-siRNA significantly decreases the apoptosis rate compared with the GINS2-siRNA group (*P*<0.05, Figure 5D).

## Discussion

Here, we studied the role and underlying molecular mechanism of GINS2 in the development of NSCLC. We found that the levels of GINS2 mRNA and protein are significantly increased in NSCLC tissues and cell lines, which is consistent with the studies of Liu et al., who reported that the level of GINS2 is up-regulated in lung adenocarcinoma [15]. Previous studies have also shown that GINS2 is highly expressed in other cancers, such as breast cancer [13], cervical cancer [18] and intrahepatic cholangiocarcinoma [19].

The GINS family is a four-subunit helicase that plays a key role in the opening of the DNA replication fork and regulation of the cell cycle [20]. During the initiation of DNA replication in eukaryotic cells, GINS2 is one of the subunits that interacts with the minichromosone maintenance (MCM) complex and Cell division control protein 45 (Cdc45) and participates in the regulation of the cell cycle and proliferation with a series of receptor growth factors and proteins [21]. Research by Rantala et al. has indicated that down-regulation of GINS2 inhibits cell proliferation and M phase progression in breast cancer cells [16]. Meanwhile, knockdown of GINS2 obviously increases the number of cells in G_2_ phase, blocks DNA synthesis and decreases proliferation in the human promyelocytic leukaemia cell line HL60 [22]. Here, we demonstrated that knockdown of GINS2 reduces cell proliferation and increases the percentage of cells in G_2_/M phase. In addition, eukaryotic cell cycle progression is tightly regulated by complexes consisting of cyclins and CDKs, in which the complex of M-phase-promoting factor (MPF) composed of cdc2 (CDK1) and cyclinB1 is a key regulator of the G_2_/M cell cycle transition [23,24]. Nocodazole, a prototypic microtubule inhibitor, can cause G_2_/M cell cycle arrest and results in strong up-regulation of cyclin B1 and CDK1 levels in human cancer cells [25,26]. Therefore, we checked the changes in cyclin-B1 and CDK1 protein after GINS2 transfection. The results indicated that knockdown of GINS2 obviously reverses the effect of nocodazole on the expression levels of CDK1 and cyclinB1 protein in NSCLC.

Uncontrolled cell proliferation and decreased apoptosis are two major characteristics of the majority of cancer cells [27]. In the study, we investigated the relationship between GINS2 knockdown and apoptosis. The data showed that down-regulation of GINS2 can promote cell apoptosis and regulate the expression levels of apoptosis-related proteins. Consistent with these results, previous studies have also shown that the knockdown of GINS2 induces apoptosis, up-regulates the pro-apoptosis-related protein Bax and down-regulates the anti-apoptosis protein Bcl-2 in other cancer cells [12,17,21].

GADD45A is the first member of the GADD45 family and a DNA-damage-induced protein [28]. GADD45A can reduce the risk of cancer by blocking the G_2_/M phase of the cell cycle, repairing DNA and promoting apoptosis [29]. Current studies have shown that abnormal expression of the GADD45A gene is closely related to lung, breast, pancreatic and prostate cancers [30–33]. The GADD45A gene is a downstream gene regulated by p53 genes and can bind directly to p53 in the third intron region of its gene [34]. P53 is one of the important tumour suppressor proteins and is one of the normally inactivated proteins in human tumour cells [35]. Zhang et al. reported that GINS2 down-regulation markedly promoted the level of p53 protein in a leukaemic cell line [12]. In agreement with those reports, our results showed that knockdown of GINS2 increased the levels of p53 and GADD45A protein, and co-transfection of GINS2 and p53-siRNA or GINS2 and GADD45A-siRNA could partially reverse the effects of GINS2 knockdown on proliferation and apoptosis in A549 cells. Together, these results support mechanistic involvement of the p53/GADD45A signalling pathway in GINS2-siRNA-mediated anti-proliferation and pro-apoptotic effects.

## Conclusion

In summary, our results demonstrate that knockdown of GINS2 inhibits cell proliferation and induces apoptosis in A549 and H450 cell lines. Suppressing expression of p53 or GADD45A reduces the effect of GINS2 knockdown. Therefore, GINS2 can be used as a potential molecular target to attenuate the progression of NSCLC.

## Abbreviations

CCK-8: Cell Counting Kit-8
cdc45: Cell division control protein 45
CDK: cyclin-dependent kinase
FCM: flow cytometry
GADD45A: growth arrest and DNA damage 45A
GINS: Go-Ichi-Ni-San
GINS2: GINS complex subunit 2
MCM: minichromosome maintenance
NSCLC: non-small-cell lung cancer
PI: propidine iodide
RT-qPCR: reverse transcription-quantitative polymerase chain reaction
siRNA: small interfering RNA
